# HE4 as a serum biomarker for the diagnosis of pelvic masses: a prospective, multicenter study in 965 patients

**DOI:** 10.1186/s12885-022-09887-5

**Published:** 2022-07-30

**Authors:** Elena Ioana Braicu, Catherine Linn Krause, Uwe Torsten, Herbert Mecke, Rolf Richter, Lars Hellmeyer, Malgorzata Lanowska, Bodo Müller, Elisa Koch, Janine Boenneß-Zaloum, Kerstin Ames, Radoslav Chekerov, Kati Hasenbein, Mathias Zimmermann, Mandy Mangler, Frank Chen, Rudolf Tauber, Jalid Sehouli

**Affiliations:** 1 Department of Gynecology with Center of Oncological Surgery, Charité – Universitätsmedizin Berlin, Freie Universität Berlin, Humboldt-Universität zu Berlin, and Berlin Institute of Health, Campus Virchow Klinikum, Augustenburger Platz 1, 13353 Berlin, Germany; 2 Department of Obstetrics and Gynecology, Stanford University, California, USA; 3 Department for Gynecology, Vivantes Klinikum Neukölln, Berlin, Germany; 4 Department for Obstetrics and Gynecology, AVK Vivantes, Berlin, Germany; 5 Department for Obstetrics and Gynecolgy, Vivantes Klinikum Friedrichshain, Berlin, Germany; 6 Department of Gynecology, Charité – Universitätsmedizin Berlin, Freie Universität Berlin, Humboldt-Universität zu Berlin, and Berlin Institute of Health, Campus Charité Mitte, Berlin, Germany; 7 Department for Gynecology, Vivantes Klinikum Kaulsdorf, Berlin, Germany; 8 Department for Gynecology, Vivantes Klinikum Humboldthain, Berlin, Germany; 9 Central Institute of Laboratory Medicine, DRK Kliniken Berlin, Berlin, Germany; 10 Charité – Universitätsmedizin Berlin, Freie Universität Berlin, Humboldt-Universität zu Berlin, and Berlin Institute of Health, Institute of Laboratory Medicine, Clinical Chemistry and Pathobiochemistry and Labor Berlin Charité, Berlin, Germany

**Keywords:** Ovarian cancer, Endometriosis, HE4, CA125, Biomarkers

## Abstract

**Background:**

To evaluate the diagnostic value of adding human epididymis protein 4 (HE4), cancer antigen 125 (CA125) and risk of malignancy algorithm (ROMA) to ultrasound for detecting ovarian cancer in patients with a pelvic mass.

**Methods:**

This was a prospective, observational, multicenter study. Patients aged > 18 years who were scheduled to undergo surgery for a suspicious pelvic mass had CA125 and HE4 levels measured prior to surgery, in addition to a routine transvaginal ultrasound scan. The diagnostic performance of CA125, HE4 and ROMA for distinguishing between benign and malignant adnexal masses was assessed using receiver operating characteristic (ROC) analysis and the corresponding area under the curve (AUC).

**Results:**

Of 965 evaluable patients, 804 were diagnosed with benign tumors and 161 were diagnosed with ovarian cancer. In late-stage ovarian cancer, CA125, HE4 and ROMA all had an excellent diagnostic performance (AUC > 0.92), whereas in stage I and II, diagnostic performance of all three biomarkers was less adequate (AUC < 0.77). In the differential diagnosis of ovarian cancer and endometriosis, ROMA and HE4 performed better than CA125 with 99 and 98.1% versus 75.0% sensitivity, respectively, at 75.4% specificity.

**Conclusions:**

ROMA and HE4 could be valuable biomarkers to help with the diagnosis of ovarian cancer in premenopausal patients in order to differentiate from endometriosis, whereas CA125 may be more adequate for postmenopausal patients.

## Highlights

• Serum biomarkers can help to distinguish benign from malignant pelvic masses

• We evaluated the diagnostic value of adding HE4, CA125 and ROMA to ultrasound for detecting ovarian cancer

• In stage III and IV ovarian cancer all three biomarkers showed excellent performance

• ROMA and HE4 performed better than CA125 in the differential diagnosis of ovarian cancer and endometriosis

## Introduction

Ovarian cancer is a leading cause of mortality from gynecological malignancies, with an estimated 313,959 new cases and 207,252 deaths worldwide in 2020 [1]. The number of cases diagnosed each year is rising with increasing life expectancy [2]. Survival rates for ovarian cancer have improved in recent decades, but the overall 5-year survival rate for all disease stages is still only around 30% [3]. The 5-year survival rate for stage I disease is far higher at 92%; however, only 15% of cases are diagnosed at this stage and there is no proven method for early detection [4].

Ovarian cancer is widely known as a ‘silent killer’ due to the lack of specific symptoms. Although most patients experience symptoms in the early stages of the disease, these are often non-specific (for example fatigue, bloating and constipation) and are associated with a number of common benign gastrointestinal, genitourinary and gynecological conditions, making early diagnosis challenging [3, 5]. Consequently, around 60% of patients have metastatic disease at the time of diagnosis [5]. The probability of a pelvic mass being benign or malignant is the key factor for steering patients to the correct institution and clinician; it has been shown that patients with ovarian cancer treated by a gynecological oncologist are more likely to receive optimal surgery compared with patients treated by gynecologists or general surgeons [6, 7]. It is therefore crucial that patients are referred to the appropriate specialist as early as possible to give the best chance of long-term survival.

Preoperative diagnostics are still one of the major challenges in the clinical routine, as all single methods have several limitations [8]. The use of serum biomarkers can help to distinguish benign from malignant masses and thus facilitate referral of patients to the most appropriate clinician. Several biomarkers have been evaluated for their potential to discriminate between benign and malignant pelvic masses, including CA125, which is a high molecular weight transmembrane mucin that is overexpressed in 80% of epithelial ovarian cancers [9], and HE4, which is a protein secreted by epithelial cells that shows increased expression in the majority of ovarian cancers [10]. In addition to its potential in first diagnosis, there are ongoing studies examining the role of HE4 in detecting recurrence [11] and even predicting optimal cytoreduction in patients with primary ovarian cancer [12, 13].

Ongoing studies suggest that early clearance of serum HE4 during chemotherapy correlates with response to chemotherapy and thus prognosis [14].

CA125 and HE4 measurements have also been combined with menopausal status to develop the risk of malignancy algorithm (ROMA), which has exhibited 94% sensitivity for ovarian cancer at 75% specificity [15]. In pivotal trials, magnetic resonance imaging was the preferred method despite the fact that in routine clinical practice ultrasound is generally the method most frequently used by gynecologists. Additionally, ultrasound is performed by an expert and has a better sensitivity and specificity than all biomarkers or algorithms [16]. However, ultrasound is very subjective and its accuracy depends heavily on the experience of the ultrasound examiner. Furthermore, while ultrasound may detect some cases of ovarian cancer at an early stage, it lacks adequate specificity and sensitivity in this setting [3].

The aim of this prospective study was to evaluate the diagnostic value of adding HE4, CA125 and ROMA to transvaginal ultrasound (TVUS) for detecting ovarian cancer in patients presenting with a pelvic mass. The differential diagnosis of endometriosis and early ovarian cancer is particularly challenging, as ultrasound features can be difficult to interpret and CA125 is usually elevated in both conditions [17]. Therefore, a sub-analysis was also performed to evaluate the ability of HE4 compared with CA125 to discriminate between endometriosis and ovarian cancer.

Patients with Borderline Tumors (BOT) were also included in the study as the differential diagnosis between epithelial ovarian cancer and BOT is difficult to be made via ultrasound [18]. Although BOT patients have a better outcome than ovarian cancer patients, comprehensive surgical staging is needed [19]. Therefore it is important to identify these patients prior to surgery and to not treat BOT as a benign disease.

## Methods

### Study design

This was a prospective, observational, multicenter study conducted at seven centers in Berlin, Germany. All centers are high volume centers which have a high expertise in the diagnosis of gynecological masses. CA125 and HE4 levels were measured prior to surgery in patients with a pelvic mass to investigate the diagnostic value of HE4, CA125 and ROMA for detecting ovarian cancer.

The study was conducted in accordance with the principles of the Declaration of Helsinki and Good Clinical Practice, and institutional review board approval was granted by the local ethics committee of Charité Universitätsmedizin Berlin (no. EA2/049/13). All patients provided written informed consent for the use of their blood samples for research purposes. Consent was given prior to ultrasound and to blood drawing.

### Patients

Eligible patients were females aged ≥18 years who were scheduled to undergo surgery for a suspicious adnexal mass and who had an available blood sample taken no more than 30 days before surgery. Patients were usually referred by their gynaecologists due to a pelvic mass for a second opinion and treatment decision. A pelvic mass was defined as suspicious when according to an investigator surgical management was indicated. Indication for surgery was physician’s choice and might include differential diagnosis, and exclusion of malignancy, or symptom control. All included patients received a standard TVUS scan. Patients were excluded if they had a previous diagnosis of ovarian cancer, had undergone bilateral oophorectomy or were known to be pregnant. Clinical data relating to family history, symptoms, menopausal status and TVUS findings were documented prior to surgery and histological results were collected after surgery. Patients who met the inclusion criteria but who subsequently did not undergo surgery were withdrawn from the study.

### Sample collection

Blood samples were collected from all enrolled patients prior to surgery, shipped to Charité Labor Berlin central lab (Berlin, Germany) within 24 hours and stored at − 20 °C. Samples were analyzed for CA125 and HE4 concentration via electrochemiluminescence immunoassay (ECLIA) using a cobas® 8000 analyzer and Roche Elecsys® CA125 and HE4 assays (Roche Diagnostics GmbH, Germany) according to the manufacturer’s instructions.

### Study endpoints

The primary study goal was the comparison of the diagnostic performance of HE4, CA125 and ROMA in combination with TVUS in detecting ovarian cancer in patients with a pelvic mass.

Secondary study endpoints included evaluation of the diagnostic performance of HE4, CA125 and ROMA in combination with TVUS in detecting stage I–II and stage III–IV ovarian cancer in patients with a pelvic mass. A sub-analysis was also performed to evaluate the sensitivity and specificity of HE4 compared with CA125 as a biomarker to discriminate between endometriosis and ovarian cancer.

### Statistical analysis

Comparison of patients with benign and malignant adnexal masses was performed using the chi-square test for categorical variables and the Mann-Whitney U test for continuous variables. The diagnostic performance of CA125, HE4 and ROMA for distinguishing between benign and malignant adnexal masses was assessed using receiver operating characteristic (ROC) analysis and the corresponding area under the curve (AUC) with 95% confidence intervals (95% CI). All analyses were performed with IBM SPSS Statistics Version 25.

## Results

### Patient demographics

In total, 1438 patients were enrolled in the study between July 2013 and December 2015. Of these, 965 patients provided laboratory and histology data. Of the 965 evaluable patients, 804 were diagnosed with benign tumors and 161 were diagnosed with ovarian cancer (including 43 patients with borderline tumors). Patient demographics and disease characteristics are summarised in Table 1. Patients who were diagnosed with ovarian cancer were generally older at diagnosis and had higher CA125 and HE4 measurements, compared with patients with benign tumors. The majority of patients with ovarian cancer had Fédération Internationale de Gynécologie et d’Obstétrique (FIGO) stage IIIC disease (32.3%), and the most common benign tumor was cystadenoma (22.3%).

**Table 1:** Patient demographics and disease characteristics

	All (***N*** = 965)	Benign tumors (***N*** = 804)	Ovarian cancer and borderline tumors (***N*** = 161)
**Median age at diagnosis, years (range)**	48 (18–86) (*n* = 853^a^)	45 (18–86) (*n* = 704^a^)	59 (27–84) (*n* = 149^a^)
**Menopausal status, n (%)**			
Premenopausal	549 (56.9)	501 (62.3)	48 (29.8)
Postmenopausal	362 (37.5)	251 (31.2)	111 (68.9)
Unknown	54 (5.6)	52 (6.5)	2 (1.2)
**Median biomarker measurements, U/mL (range)**			
CA125	18.4 (2–11,616)	16.1 (2–6522)	159.2 (6.1–11,616)
HE4	54.52 (13.19–5039)	51.61 (13.19–5039)	149.50 (28.28–4676)
**FIGO stage, n (%)**			
IA	44 (4.6)	–	44 (27.3)
IB	3 (0.3)	–	3 (1.9)
IC	12 (1.2)	–	12 (7.5)
IIA	3 (0.3)	–	3 (1.9)
IIB	2 (0.2)	–	2 (1.2)
IIC	4 (0.4)	–	4 (2.5)
IIIA	4 (0.4)	–	4 (2.5)
IIIB	7 (0.7)	–	7 (4.3)
IIIC	52 (5.4)	–	52 (32.3)
IV	30 (3.1)	–	30 (18.6)
**Benign tumor type, n (%)**			
Cystadenoma	179 (18.5)	179 (22.3)	–
Functional cyst	165 (17.1)	165 (20.5)	–
Endometriosis	109 (11.3)	109 (13.6)	–
Cystadenofibroma	47 (4.9)	47 (5.8)	–
Dermoid cyst	44 (4.6)	44 (5.5)	–
Inclusion cyst	44 (4.6)	44 (5.5)	–
Teratoma	34 (3.5)	34 (4.2)	–
Endometrioid tumor	20 (2.1)	20 (2.5)	–
Tubo-ovarian abscess	17 (1.8)	17 (2.1)	–
Fibroma	15 (1.6)	15 (1.9)	–
Benign Brenner tumor	5 (0.5)	5 (0.6)	–
Other	124 (12.8)	125 (15.2)	–

*FIGO* Fédération Internationale de Gynécologie et d’Obstétrique, *HE4* Human epididymis protein 4

^a^Patients aged < 18 years were not included

### Diagnostic performance of CA125, HE4 and ROMA in detecting ovarian cancer

**Fig. 1:**
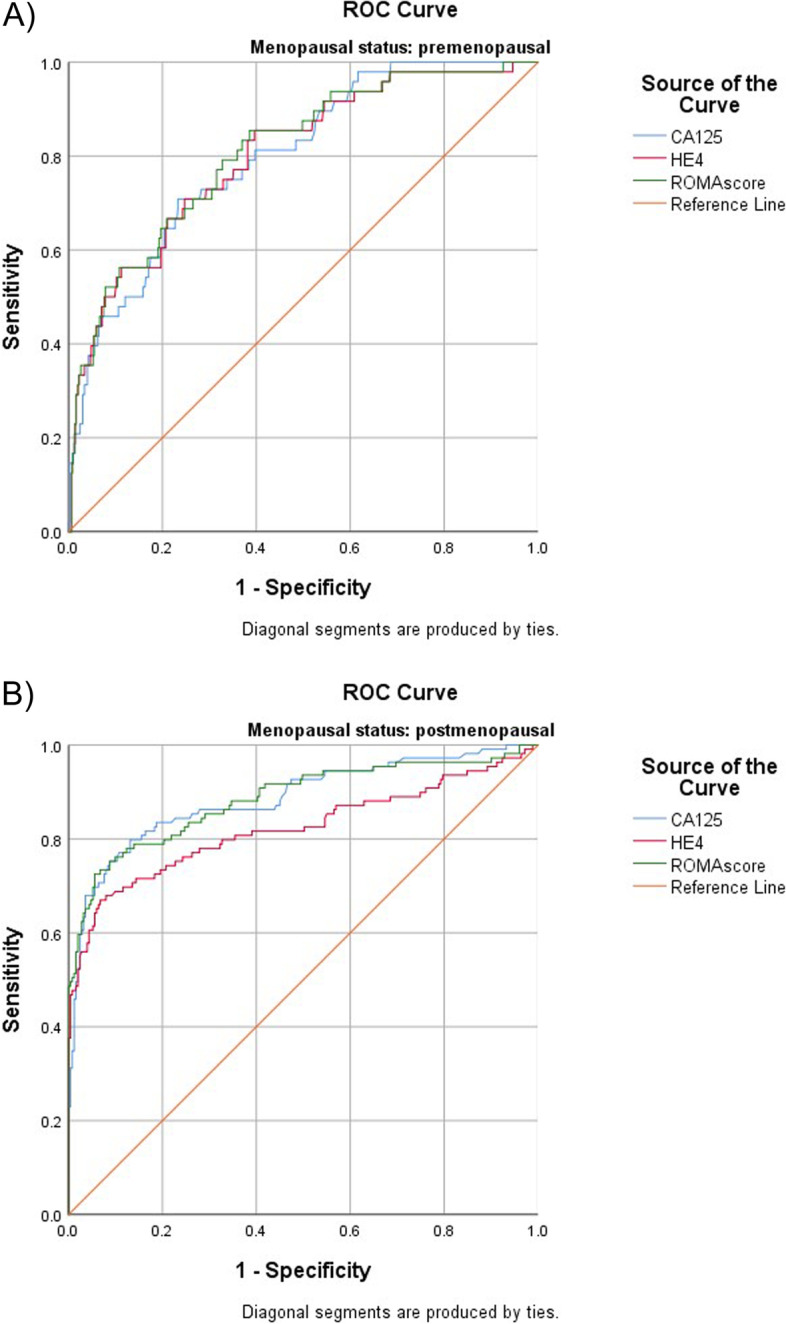
Diagnostic performance of CA125, HE4 and ROMA for detection of ovarian cancer in premenopausal (**A**) and postmenopausal (**B**) patients

In premenopausal patients HE4, CA125 and ROMA showed a comparable performance (Fig. 1A). The AUC for HE4 was 0.80 (95% CI 0.74–0.87) versus 0.80 (95% CI 0.74–0.86) for CA125 versus (AUC 0.81 [95% CI 0.74–0.87]) for ROMA. In postmenopausal patients, CA125 and ROMA performed slightly better than HE4 (Fig. 1B), with AUCs of 0.89 (95% CI 0.84–0.93) for CA125 and 0.86 (95% CI 0.84–0.93) for ROMA, compared with 0.82 (95% CI 0.77–0.88) for HE4.

### Diagnostic performance of CA125, HE4 and ROMA in detecting early-stage ovarian cancer

**Fig. 2:**
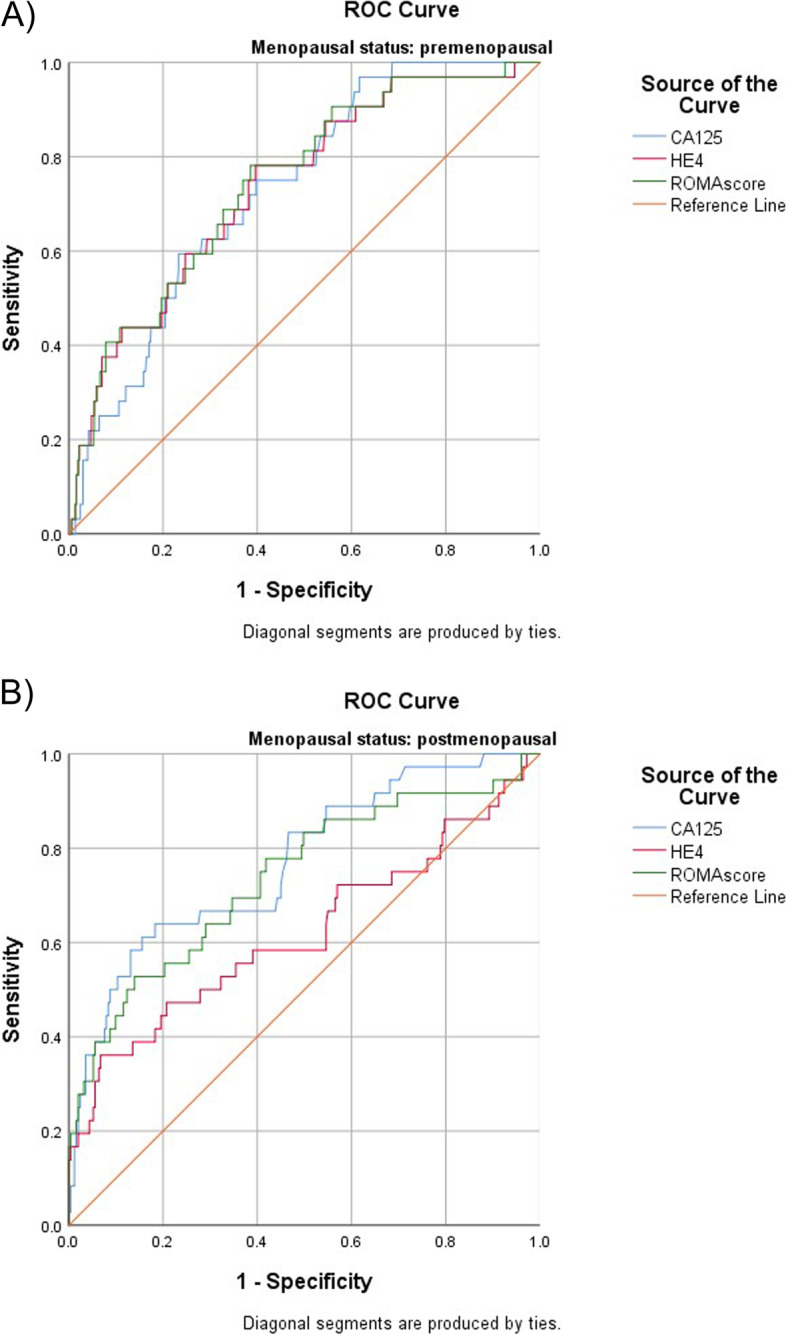
Diagnostic performance of CA125, HE4 and ROMA for detection of Stage I and Stage II ovarian cancer in premenopausal (**A**) and postmenopausal (**B**) patients

In premenopausal patients, performance of all biomarkers was nearly equivalent. AUCs were 0.73 (95% CI 0.65–0.81) for CA125, 0.74 (95% CI 0.65–0.82) for HE4 and 0.74 (95% CI 0.66–0.83) for ROMA (Fig. 2A). In postmenopausal patients, CA125 and ROMA performed slightly better than HE4 in the detection of stage I and stage II disease (Fig. 2B). AUCs were 0.77 (95% CI 0.68–0.86) for CA125 and 0.74 (95% CI 0.64–0.83) for ROMA, compared with 0.62 (95% CI 0.50–0.74) for HE4.

### Diagnostic performance of CA125, HE4 and ROMA in detecting late-stage ovarian cancer

**Fig. 3:**
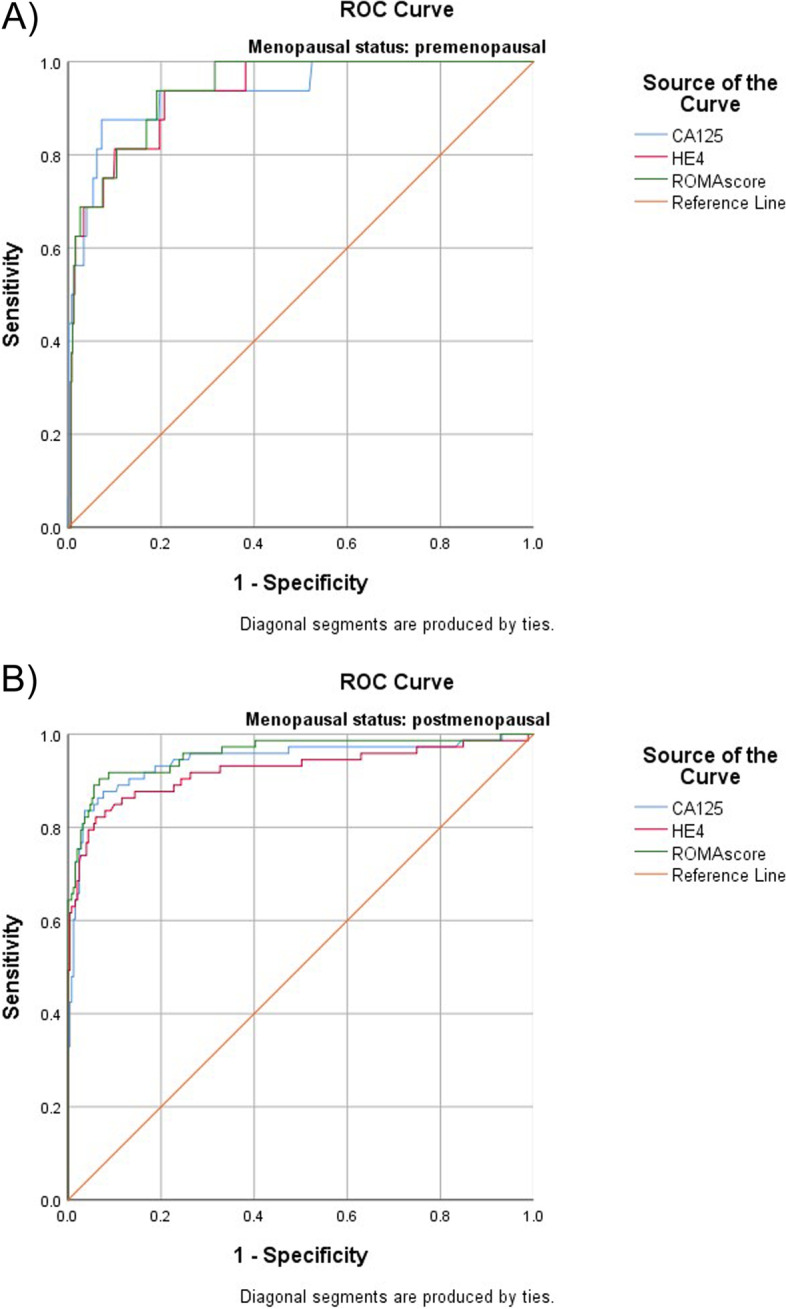
Diagnostic performance of CA125, HE4 and ROMA for detection of Stage III and Stage IV ovarian cancer in premenopausal (**A**) and postmenopausal (**B**) patients

In premenopausal patients, performance of all biomarkers was nearly equivalent (Fig. 3A). AUCs were 0.94 (95% CI 0.87–1.00) for CA125 and 0.93 (95% CI 0.88–0.98) for HE4 and 0.94 for ROMA [95% CI 0.90–0.98]). Similarly, in postmenopausal patients, ROMA, CA125 and HE4 performed nearly equivalent in detecting late-stage ovarian cancer (AUCs 0.96 [95% CI 0.93–0.99], 0.94 [95% CI 0.91–0.98] and 0.92 [95% CI 0.88–0.97], respectively) (Fig. 3B).

### Diagnostic performance of HE4 compared with CA125 to distinguish between ovarian cancer and endometriosis

**Fig. 4:**
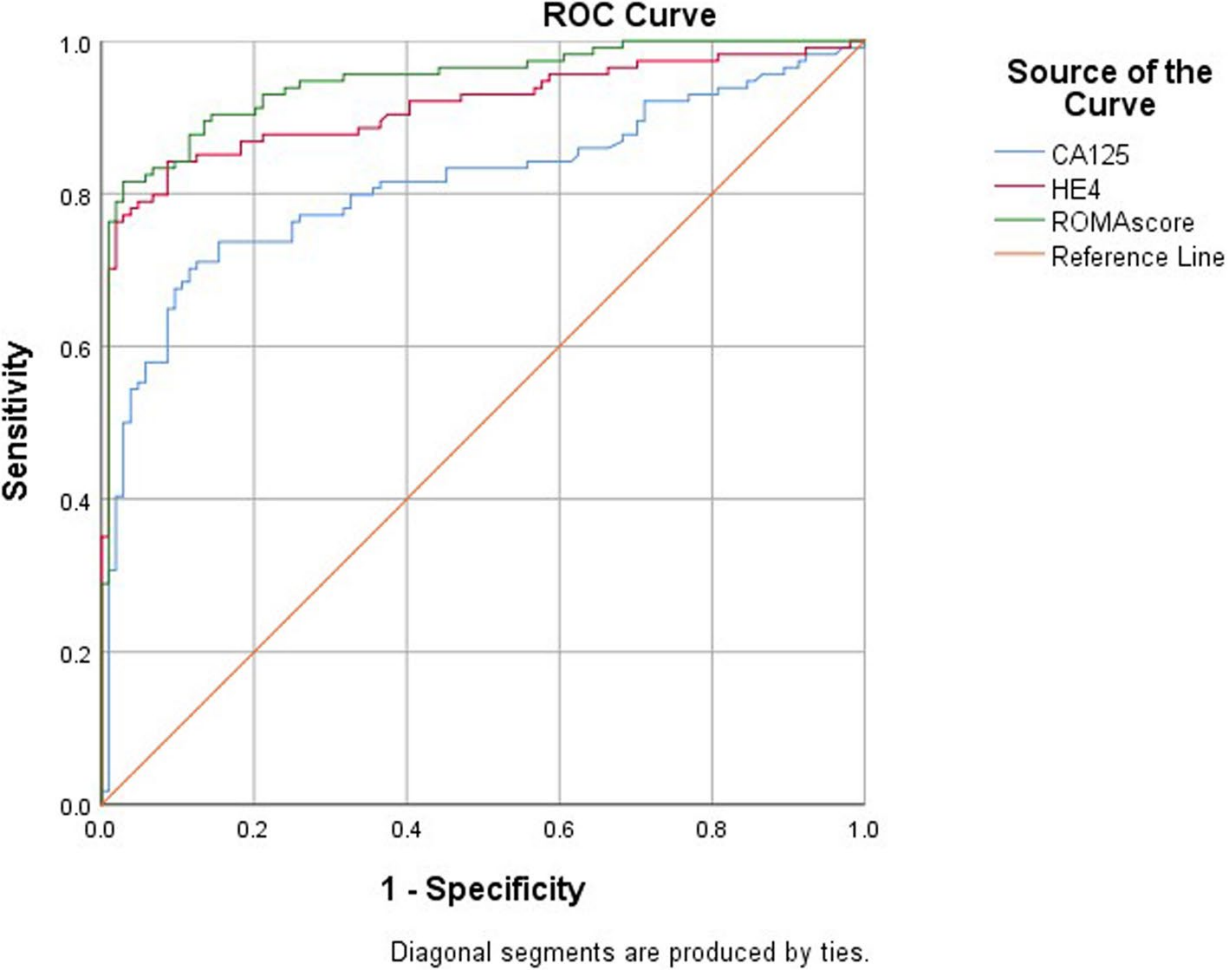
Diagnostic performance of CA125, HE4 and ROMA for the differential diagnosis of ovarian cancer and endometriosis

In the sub-analysis to evaluate the sensitivity and specificity of HE4 compared with CA125 as a biomarker to discriminate between endometriosis and ovarian cancer, 114 patients were diagnosed with epithelial ovarian cancer and 104 patients were diagnosed with endometriosis. In these patients, HE4 performed better than CA125 in the differential diagnosis of ovarian cancer and endometriosis (Fig. 4). AUCs were 0.91 (95% CI 0.87–0.95) for HE4 and 0.81 (95% CI 0.75–0.87) for CA125. ROMA outperformed HE4 and CA125 with an AUC of 0.95 (95% CI 0.92–0.98). At a predefined specificity of 75.4%, the respective sensitivities for CA125, HE4 and ROMA were 75.0, 98.1 and 99.0%.

### Diagnostic performance of CA125, HE4 and ROMA to distinguish between early ovarian cancer and endometriosis

**Fig. 5:**
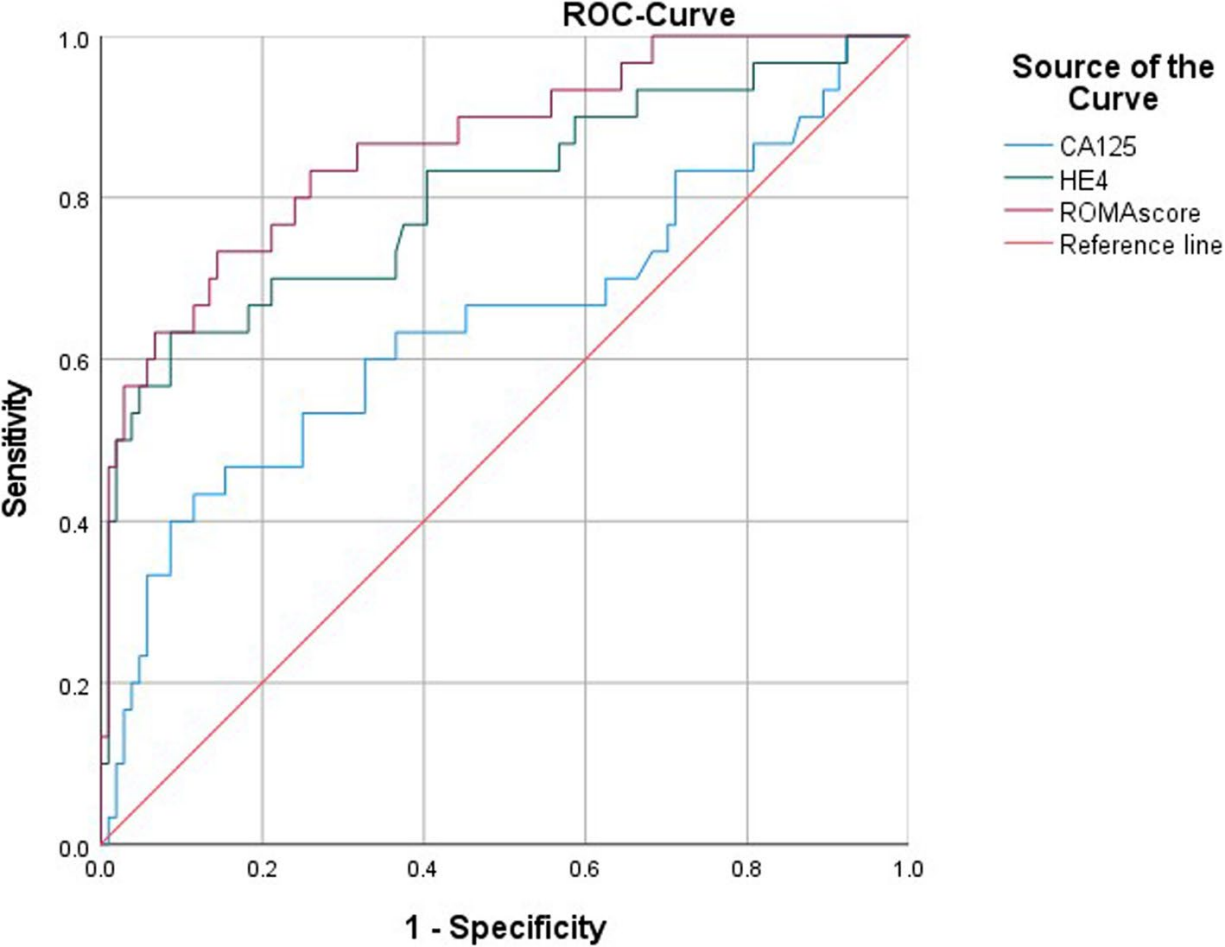
Diagnostic performance of CA125, HE4 and ROMA for the differential diagnosis of early stage ovarian cancer and endometriosis

In the sub-analysis to evaluate the sensitivity and specificity of CA125 and HE4 compared with ROMA to discriminate between endometriosis and early ovarian cancer, 30 patients were diagnosed with early epithelial ovarian cancer FIGO I-II and 104 patients were diagnosed with endometriosis. The best sensitivity and specificity in identifying early stage ovarian cancer from endometriosis patients was shown by the ROMA algorithm (Fig. 5). The results showed an AUC of 0.647 (*p* = 0.015, 95%CI: 0.52–0.77), 0.804 (*p* < 0.001, 95%CI: 0.703–0.903) and 0.865 (*p* < 0.001, 95%CI: 0.788–0.942) for CA125, HE4 and ROMA, respectively.

## Discussion

Serum biomarkers can help to distinguish benign tumors from malignant pelvic masses, ensuring that patients are quickly directed to the most appropriate clinician [3]. Patients who present with features suggestive of a benign pelvic mass can be managed successfully and cost efficiently by a gynecologist or a general surgeon, whereas patients with features suggestive of malignancy should be referred to a specially-trained and experienced gynecological oncologist for evaluation, as management of ovarian cancer by a specialist significantly improves patient outcomes [3].

It is well established that serum CA125 levels can be elevated in patients with ovarian cancer, but this biomarker has a low sensitivity in the early stages of disease and raised CA125 levels have also been observed in other physiological or pathological conditions, including menstruation, pregnancy, endometriosis and inflammatory diseases of the peritoneum [17]. HE4 has also been shown to be a potential diagnostic biomarker for ovarian cancer. It is overexpressed in ovarian cancer [20] and when compared with multiple biomarkers (including CA125), HE4 demonstrated the highest sensitivity for distinguishing ovarian cancer from benign disease (72.9% at 95% specificity) [15]. CA125 and HE4 have also been shown to have a potential role in predicting recurrence after treatment, survival after a recurrence, and surgical outcome [21–23] and CA125 has demonstrated diagnostic value in borderline tumors of the ovary [24].

To our knowledge, we report the largest prospective study which combined biomarker data with vaginal ultrasound. We demonstrated that in late-stage ovarian cancer, all of the evaluated markers (CA125, HE4 and ROMA) had an excellent diagnostic value independent of menopausal status (all AUCs > 0.92). This data is in line with other meta- analyses which report AUCs ranging from 0.78 to 0.90 for CA125, 0.89 to 0.93 for HE4 and 0.84 to 0.96 for ROMA [25].

However, in patients with early-stage cancer, the diagnostic value was less accurate (all AUCs < 0.75). In premenopausal patients with stage I or II disease, no significant difference was observed between the three parameters. In postmenopausal patients, HE4 alone was outperformed by CA125 and ROMA (AUC 0.62 versus 0.77 and 0.74, respectively).

This reflects the dilemma that early-stage ovarian cancer is extremely difficult to diagnose by imaging or tumor markers and that a screening or early detection program is not at hand. Additionally, the cost-benefit relationship favors the application of vaginal ultrasound.

In postmenopausal patients, CA125 and ROMA consistently outperformed HE4 alone, indicating that ROMA and CA125 may be the biomarkers of choice for detecting ovarian cancer in this subgroup of patients. Our findings suggest that in young patients with unclear imaging results, HE4 and ROMA could be used as biomarkers to provide additional information on the likelihood of ovarian cancer being present.

Previous reports have shown HE4 to be more reliable than CA125 in diagnosing ovarian cancer [26–29] and in a study of multiple biomarkers, HE4 was the best single marker for stage I disease [30]. In line with our findings, a recent multicenter Italian study evaluating biomarker diagnostic performance in 387 patients reported sensitivities of 69.6% for HE4 versus 65.2% for CA125 in premenopausal patients, and 78% for HE4 versus 88% for CA125 in postmenopausal patients (all at 98% specificity) [26], further suggesting that HE4 may be most informative when used in younger patients. Combining CA125 with HE4 has been reported to produce higher sensitivity (76.4% versus 72.9% for HE4 alone), suggesting that a combination of the two biomarkers may provide a more accurate prediction for malignancy than either biomarker alone [30]. Indeed, high AUCs have been reported with the combination of HE4 and CA125, varying from 0.91 to 0.96 [30, 31]. However, a recent study looking at combined use of these biomarkers specifically in postmenopausal women reported no added value when HE4 was added to CA125, again suggesting that HE4 is best used in premenopausal patients [31].

Several algorithms have been established which combine age, menopausal status, imaging data and serum biomarker measurements to estimate the risk of a mass being malignant. These include: the risk of malignancy index (RMI), which combines ultrasound, menopausal status and serum CA125 levels; OVA1, which combines data from imaging, menopausal status and CA125 levels with four additional biomarkers (apolipoprotein A1, transthyretin, transferrin and β2-macroglobulin); and ROMA, which combines menopausal status with CA125 and HE4 levels [32–34]. In this study, we used ROMA, which has a reported sensitivity of 92% in postmenopausal women and 77% in premenopausal women (both at 75% specificity) [34]. Some studies have confirmed the predictive value of ROMA in the detection of ovarian cancer [28, 35, 36], while others have reported that ROMA performs no better than either CA125 or HE4 alone [37, 38]. In the current study, we observed that the combination of HE4 levels, CA125 levels and menopausal status in the ROMA score outperformed either biomarker alone in premenopausal patients in terms of overall, early-stage and late-stage ovarian cancer. A recent study reported that the predictive power of ROMA is not significantly better than that of HE4 in premenopausal women (AUC 0.731 versus 0.732, respectively) or than that of CA125 in postmenopausal women (AUC 0.871 versus 0.888) [39].

In our sub-analysis of patients diagnosed with epithelial ovarian cancer or endometriosis, HE4 showed a higher sensitivity than CA125 in distinguishing between the two conditions (98.1% versus 75.0%, respectively, at 75.4% specificity). This reflects evidence from previous studies showing HE4 to be a better biomarker in this setting, with similar sensitivity to CA125 (82–87% for HE4 versus 82–90% for CA125) but higher specificity (100% for HE4 versus 49–70% for CA125) [40, 41]. The better performance of HE4 is due to the fact that CA125 levels are often elevated in both ovarian cancer and endometriosis, whereas HE4 levels remain stable in endometriosis [42]. In our study, ROMA outperformed both HE4 and CA125 in this setting, with 99.0% sensitivity at 75.4% specificity. This was comparable in all ovarian cancer stages and early ovarian cancer FIGO I-II compared to endometriosis.

More research is needed into the use of HE4 for the detection of ovarian cancer. Notably, cut-off values need to be validated, with separate defined values for premenopausal and postmenopausal patients. Studies to date have not provided a universal HE4 reference range for healthy women from different populations, and values used by different laboratories vary [43]. It is also noteworthy that unlike CA125, HE4 levels may be increased by smoking and decreased by the use of oral contraception [44, 45]. Therefore, these lifestyle factors should be taken into account when interpreting HE4 measurements.

In the present study we did not systematically analyse the dynamics of the biomarkers; therefore, future trials should include preoperative analyses of CA125 and HE4 to evaluate whether this additional information can increase the ability of these biomarkers to discriminate between benign and malignant pelvic masses.

In conclusion, the results presented here add to existing evidence that ROMA and HE4 could be valuable biomarkers to assist with the diagnosis of ovarian cancer in premenopausal patients. The use of HE4 measurements and ROMA calculation in this setting may help to facilitate referral of patients to the appropriate specialist to give the best chance of optimal treatment and long-term survival. In postmenopausal patients, CA125 may be the most accurate marker.

## Data Availability

The data that support the findings of this study are available within TOC Biobank but restrictions apply to the availability of these data, which were used under license for the current study, and so are not publicly available. Data are however available from the authors upon reasonable request.

## Abbreviations

HE4: Human epididymis protein 4
CA125: Cancer antigen 125
ROMA: Risk of malignancy algorithm
AUC: Area under the curve
TVUS: Transvaginal ultrasound
ECLIA: Electrochemiluminescence immunoassay
ROC: Receiver operating characteristic
FIGO: Fédération Internationale de Gynécologie et d'Obstétrique
BOT: Borderline Tumor
